# Ophthalmic surgeries on *post mortem* porcine eyes with picosecond ultrashort laser pulses

**DOI:** 10.3389/fmed.2024.1345976

**Published:** 2024-02-08

**Authors:** Michael Körber, Jakob Fellinger, Milan Fritsche, Andreas Giese, Konstantina Kostourou, Daniel Kopf, Manfred Kottcke, Francesco Luciani, Josef M. Schmidbauer, Jonathan Wenk, Bernd Braun

**Affiliations:** ^1^Applied Mathematics, Physics and Humanities, Nuremberg Institute of Technology, Nuremberg, Germany; ^2^Paracelsus Medical University, Nuremberg, Germany; ^3^MONTFORT Laser GmbH, Götzis, Austria; ^4^NANEO Precision IBS Coatings GmbH, Lindau, Germany; ^5^Clinic of Ophthalmology, Klinikum Nürnberg Nord, Nuremberg, Germany

**Keywords:** iridotomy, capsulotomy, SLT, picosecond laser, shock waves, glaucoma, cataract

## Abstract

**Purpose:**

This work demonstrates significant advantages in ophthalmic surgeries through the use of picosecond ultrashort laser pulses instead of state-of-the-art nanosecond laser pulses. These ultrashort lasers shall serve as universal tools more effectively combining advantages of high precision, low impact and economic advantages compared to existing instruments.

**Methods:**

As samples, we used post-mortem porcine eyes on which we performed the experiments with both picosecond and nanosecond lasers. Performed surgeries were laser iridotomy, (post-) cataract treatment/capsulotomy and selective laser-trabeculoplasty. Pulse widths were between 12 ps and 220 ns with pulse energies between 30 μJ and 10 mJ at 532 nm and 1,064 nm. Additionally, we investigated accompanying shock waves, cavitation bubbles, and heat effects during the ablation processes.

**Results:**

For all surgeries, significant differences were observed between picosecond and nanosecond pulses: It was possible to scale the pulse energy down to 10 of microjoules rather than requiring millijoules, and resulting tissue ablations are much more precise, more deterministic and less frayed. The shock wave and cavitation bubble investigation revealed major differences in pressure between picosecond pulses (0.25 MPa, 50 μJ) and nanosecond pulses (37 MPa, 5 mJ). The heat input during ablation could be lowered by two orders of magnitude.

**Conclusion:**

Picosecond ultrashort laser pulses show substantial benefits for several ophthalmic surgeries, with regard to ablation precision, shock wave generation and heat input. They are better than state-of-the-art ophthalmic nanosecond lasers in all aspects tested.

## 1 Introduction

In ophthalmology, laser sources are commonly used for non-invasive/incisionless or minimally invasive surgeries. These lasers are mainly neodymium doped yttrium-aluminum-garnet (Nd:YAG) lasers. They emit comparatively long pulse widths in the nanosecond (ns) range that lead to a non-negligible heat input during surgical tissue ablation. The energy input then causes immediate boiling, expansion and evaporation of the tissue. This results in acoustic shock waves and non-deterministic tissue ablation with collateral damage of surrounding tissue. Consequently, the ablation achieved is much larger than the actual laser spot, which can lead to side effects and additional clinical patterns such as ghost images, iris bleeding, corneal endothelial damage or retinal detachment. Furthermore, the tissue ablation with ns laser sources depends on the linear absorption properties of the tissue and therefore on the color of the eye (1–7). However, such unintended results can be counteracted by using ultrashort pulses (USP) (8–13). The main advantage of these pulses—which have a pulse width of a few picoseconds or less—is to be near the regime of cold material ablation. This physical effect results from the very short pulse width and the associated high peak intensity. Due to the high peak intensities, ionization and plasma formation occur through multi-photon processes when a single laser pulse interacts with the tissue. This interaction time is too short to transfer energy to the atomic lattice of the material, as the heat diffusion time in the lattice is much longer than the duration of the laser pulse. This results in material removal with very little heat input. Another advantage of cold material ablation is that the non-linear absorption properties of the material is predominant, which is why the ablation is almost independent of the material properties in terms of linear absorption, reflection and transmission (14–17). Consequently, USP lasers should ablate tissue very precisely and deterministically independent of eye color (10, 18–23). However, currently available ultrashort pulse lasers, such as those used for LASIK (laser *in situ* keratomileusis), are very expensive and therefore economically not appropriate for small surgeries such as laser iridotomy and post-cataract treatment. For this reason, ns laser sources are state-of-the-art in ophthalmology.

This paper discusses the use of an ultrashort pulse laser source emitting in the picosecond regime for ophthalmic surgeries and evaluates the potential benefits of these lasers, particularly for iridotomy, capsulotomy/post-cataract treatment and selective laser-trabeculoplasty (SLT). This research was motivated by ultra-compact Q-switched picosecond lasers recently becoming available, combining the advantages of the low price of currently used ns laser sources with cold material ablation capabilities of high-end femtosecond lasers (24, 25).

Laser iridotomy as treatment of narrow angle glaucoma involves piercing the iris next to the Schlemm’s canal to create a new floating path for the eye fluid. This piercing is done with a series of laser pulses that are applied to the tissue via an ophthalmic slit lamp laser system. The energy ranges from 1 to 10 mJ per pulse for the ns laser sources currently in use. The same laser source is used for post-cataract treatment, where the posterior lens capsule is opened with laser pulses applied in a cross-shaped pattern. Both treatments may lead to adverse effects and additional clinical patterns such as frayed edges, collateral damage and strong shock wave generation. The latter takes place, because tissue ablation with laser sources is accompanied by a laser-induced optical breakdown in the laser focus (8). Such breakdown leads to plasma formation and the generation of shock waves caused by cavitation formation inside the material (tissue). The cavitation bubble then oscillates as long as enough energy is available in the breakdown region. Here, the initial breakdown and every cavitation bubble collapse lead to a separate shock wave. Therefore, several shock waves are generated per laser pulse and alter the mechanisms of laser ablation by affecting tissue adjacent to the target. This leads to bigger ablation areas and—if the shock waves are strong enough—to additional undesired side effects such as retinal detachment after iridotomy or (post-) cataract surgery (2, 5, 26–28). Therefore, it is necessary to ablate tissue with as low pulse energy as possible. With ultrashort pulse ps laser sources, the same treatment can be performed with much lower energy input. Furthermore, it can be shown that USP lasers lead to much less intense shock wave formation than ns lasers due to the aforementioned cold material ablation (8, 9, 29–33). The same principle can be applied to SLT, whereby the trabecular meshwork (TM) is irradiated with a 532 nm laser source. Here, the laser pulses ablate some TM tissue and also stimulate the remaining TM tissue so that previously restricted immune reactions are made viable again. Furthermore, the lasers can also be used to penetrate the TM completely down to the Schlemm’s canal to create a drainage (34–36). Both surgical methods can be performed with lower pulse energy and higher precision by using ultrashort pulse laser sources.

To investigate and prove our hypotheses, the research was executed as follows: First, the method and its application in laser iridotomy, post-cataract treatment/capsulotomy and SLT are described. Then, a quantitative analysis of shock waves associated with the treatment is presented. Finally, this paper investigates heat input of ultrashort laser pulses on biological tissue (porcine iris) and on aluminum plates, to gain a better understanding of the ablation processes and heat effects inside the tissue.

## 2 Materials and methods

This section describes the laser sources and methods used for the different experiments, namely ophthalmic surgeries, shock wave and cavitation bubble examination, and heat investigation.

### 2.1 Laser sources

Different laser beam sources are used for the experiments, emitting sech^2^ (picosecond laser) and gaussian (nanosecond lasers) shaped pulses with pulse widths between 12 ps and 220 ns and pulse energies between 30 μJ and 10 mJ, optionally at 1064 nm and 532 nm wavelength. Here, the nanosecond lasers are used as reference laser sources for state-of-the-art results. For all beam sources, the pulse energy can be adjusted manually with a polarizing beam splitter and a wave-plate up to the specific maximum. The following list describes the laser sources in more detail:

Laser 1: USP laser source with 12 ps pulse width: USP laser system consisting of a passively mode-locked Nd:YVO_4_ (neodymium-doped yttrium orthovanadate) oscillator and an Nd:YVO_4_ regenerative amplifier (37). The laser system emits a 1,064 nm pulse train with an adjustable pulse repetition rate between 1 Hz and 300 kHz, a pulse energy of up to 164 μJ, and a pulse width of 12 ps. The laser output can additionally be frequency doubled with a KTP (potassium titanyl phosphate—KTiOPO_4_) nonlinear crystal, leading to a wavelength of 532 nm with a pulse energy conversion efficiency of up to 70%.

Laser 2: ns laser source with 1.6 ns pulse width: 1.6 ns, 20 Hz quasi-continuous-wave (QCW) passively Q-switched (PQS) Nd:YAG (neodymium-doped yttrium aluminum garnet) laser source with pulse energies up to 7 mJ at 1064 nm from *MONTFORT Laser GmbH, Götzis, Austria*.

Laser 3: ns laser source with 8 ns pulse width: 8 ns, 10 Hz actively Q-switched laser source with pulse energies up to 50 mJ at 1064 nm from *MONTFORT Laser GmbH, Götzis, Austria*. The laser output can additionally be frequency doubled with a KTP nonlinear crystal, leading to a wavelength of 532 nm.

Laser 4: ns laser source with adjustable pulse width: 20 W, 1062 nm pulsed fiber master oscillator power amplifier (MOPA) laser from *JENOPTIK Laser GmbH, Jena, Germany (‘JenLas^®^fiber ns 20 advanced single mode’)*. It emits in continuous wave (cw) operation or pulsed operation with pulse widths between 15 ns and 220 ns with maximum pulse energies between 68 and 570 μJ, respectively.

### 2.2 Method for ophthalmic surgeries

In contrast to ophthalmic surgical methods with special eye attachment optics/system, the different laser beams are focused on the samples via the following beam delivery system: This system consists of a 60 mm plano convex focusing lens, an x-y-z-translation stage for sample positioning, two laser diodes for laser focus alignment (*FP-D-532-1-C-F* from *Laser Components GmbH, Olching, Germany*), and a stereo microscope as visualization device for real-time observation and magnification (*Stemi 508 trino* from *Carl Zeiss Microscopy GmbH, Oberkochen, Germany*). This set-up is based on slit-lamp laser beam delivery systems such as used in clinics and is similar in function. For part of the capsulotomies and SLT, a laser scanner set-up is used, consisting of a two-axis laser scanner head (*SS-II-15 [Y] D2* from *Raylase GmbH, Wessling, Germany*) with the F-Theta flat field objective *S4LFT3046/328* (*Sill Optics GmbH & Co. KG, Wendelstein, Germany*) with a focal length of 50 mm. Since these components of the set-ups are designed for the near infrared (NIR), a second set-up for 532 nm was built, using another 60 mm plano convex focusing lens and the scanner head *SS-IIE-15 [DY] D2* (*Raylase GmbH, Wessling, Germany*) with the F-Theta flat field objective *S4LFT4066/292* (*Sill Optics GmbH & Co. KG, Wendelstein, Germany*) with a focal length of 67.2 mm. Focusing results in a focal spot diameter of about 25 μm for all focusing set-ups. This leads to fluences of 8 J/cm^2^ (12 ps, 50 μJ) and 800 J/cm^2^ (8 ns, 5 mJ), and peak intensities of 587 GW/cm^2^ and 94 GW/cm^2^, respectively.

The tissue samples used are *post mortem* porcine eyes, which are processed about 4 h after excision. The eyes are moistened with water before, during and after all surgeries. The iridotomy and capsulotomy surgeries were performed as open sky surgeries (with prior cornea removal) for better comparability without the influence of the cornea. SLT surgeries were performed in two ways: First, on a bisected porcine eye (removed lens) with the slit-lamp set-up to focus the laser beams directly on the trabecular meshwork (TM) without the necessity of a clinically used gonioscope. Second, on an exposed trabecular meshwork with the scanner set-up. For this, the eyes were prepared in such a way that the cornea, about 2 mm of the sclera around the cornea, and the TM were preserved. This is comparable to pieces of human donor corneas, where a part of the sclera and the TM are also still attached. The lasers can then be focused directly on the TM from the posterior side. Visualization, examination and measurement of the results are carried out using high-resolution light microscopy and scanning electron microscopy (SEM). For the latter, a special sample preparation and drying method is applied to the tissue samples that avoid water inside the tissue to boil and expand under vacuum in the plasma coating machine and the SEM. This would lead to warpage/deformation/distortion and destruction of the tissue. The preparation method starts with 1,5-pentanedial (glutaraldehyde solution 50% in water (5.6 mol); *Sigma-Aldrich, St. Louis, United States*) for protein linkage and tissue fixation. The tissue is then buffered with a phosphate buffer of pH = 7.0 (*Bernd Kraft, Duisburg, Germany*) and subsequently placed in an alcoholic solution in ascending concentration (start: 20%/80% ethanol/water; end: 100% ethanol; gradation: 10% ethanol—water: purified water; ethanol: Ethanol absolute ≥99.5% from *VWR International, Radnor, United States*). The last step before air drying is soaking the tissue in 1,1,1-Trimethyl-N-(trimethylsilyl)silanamine (hexamethyldisilazane, HMDS; *Sigma-Aldrich, St. Louis, United States*). The samples of the exposed TM for SLT are soaked in pure ethanol only and subsequently air dried, because the cornea and sclera lead to a high stiffness even without fixation. Some of the capsule samples are strictly air dried during compression to avoid warpage/deformation/distortion.

All samples can then be metal-coated (metal: gold/palladium—*SC502-314B* from *Quorum Technologies, Laughton, United Kingdom*) and visualized by SEM. For this, the plasma coating machine *Q150R ES* (*Quorum Technologies, Laughton, United Kingdom*) and the *SEM JSM 6510* (*Jeol Ltd., Tokyo, Japan*) are used. Light microscope images were taken using the incident light microscope *Axio Lab.A1*, the transmitted light microscope *Primo Star* and the stereo microscope *Stemi 508 trino*, all from *Carl Zeiss AG, Oberkochen, Germany*.

In total, the research was performed on about 400 porcine eyes with over 1,000 surgeries (approx. 800 iridotomies, 25 capsulotomies, 200 SLTs).

### 2.3 Method for shock wave and cavitation bubble investigation

**Figure 1:**
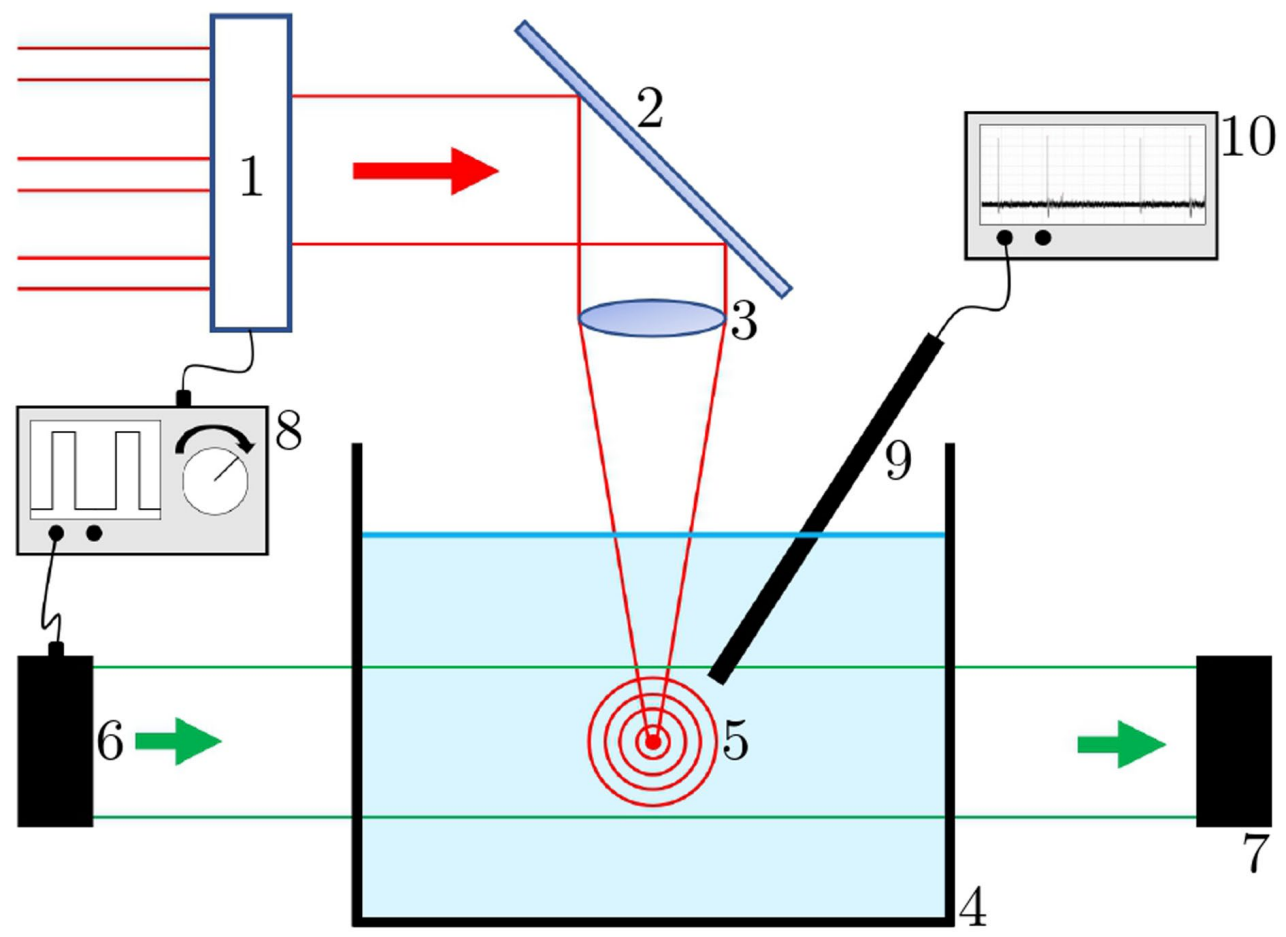
Shock wave measurement set-up. 1: switch for different laser sources; 2: laser mirror; 3: focusing lens; 4: water bath; 5: laser induced breakdown with shock waves; 6: illumination source; 7: camera; 8: function generator; 9: needle hydrophone; 10: oscilloscope.

Shock waves generated by laser pulses were investigated using the following method and set-up (see Figure 1): The different laser sources are focused via a laser mirror and an *f* = 40 mm aspherical plano-convex lens in a basin (74 mm × 74 mm × 33 mm; l × w × h) filled with distilled water. Focusing results in a diameter of about 10 μm, leading to fluences of 50 J/cm^2^ (12 ps, 50 μJ) and 5 kJ/cm^2^ (8 ns, 5 mJ), and peak intensities of 5.5 TW/cm^2^ and 587 GW/cm^2^, respectively. The laser input results in an optical breakdown and shock waves, which are visualized using a streak camera system. The field of observation has a diameter of approximately 20 mm, roughly corresponding to the distance between laser focus and retina.

The light source of the system, a collimated pulsed green laser diode *PLT5 510 B1-3* (*OSRAM GmbH, München, Germany*), is synchronized to the pulse repetition rate of the breakdown laser. The phase delay between the laser pulse and the illumination pulse can be adjusted manually with a ns-increment to illuminate different shock wave propagation points. Both synchronization and delay adjustment are performed with a waveform generator (*33500B* from *Agilent Technologies, Inc., Santa Clara, United States*). Recording is done with a digital camera (*RX100* from *Sony, Tokyo, Japan*). In addition to the photographs, a 2 mm diameter needle hydrophone with attenuator, pre-amplifier and DC-coupler (*Precision Acoustics Ltd., Dorchester, United Kingdom*) is placed next to the breakdown below the water surface. It serves as a receiver of the shock wave fronts. For visualization and measurement, it is connected to an oscilloscope (*MSO64 from Tektronix, Inc., Beaverton, United States*, 6 GHz bandwidth). The result of this measurement is a quantitative intensity profile of the shock waves as a function of the laser parameters.

**Figure 2:**
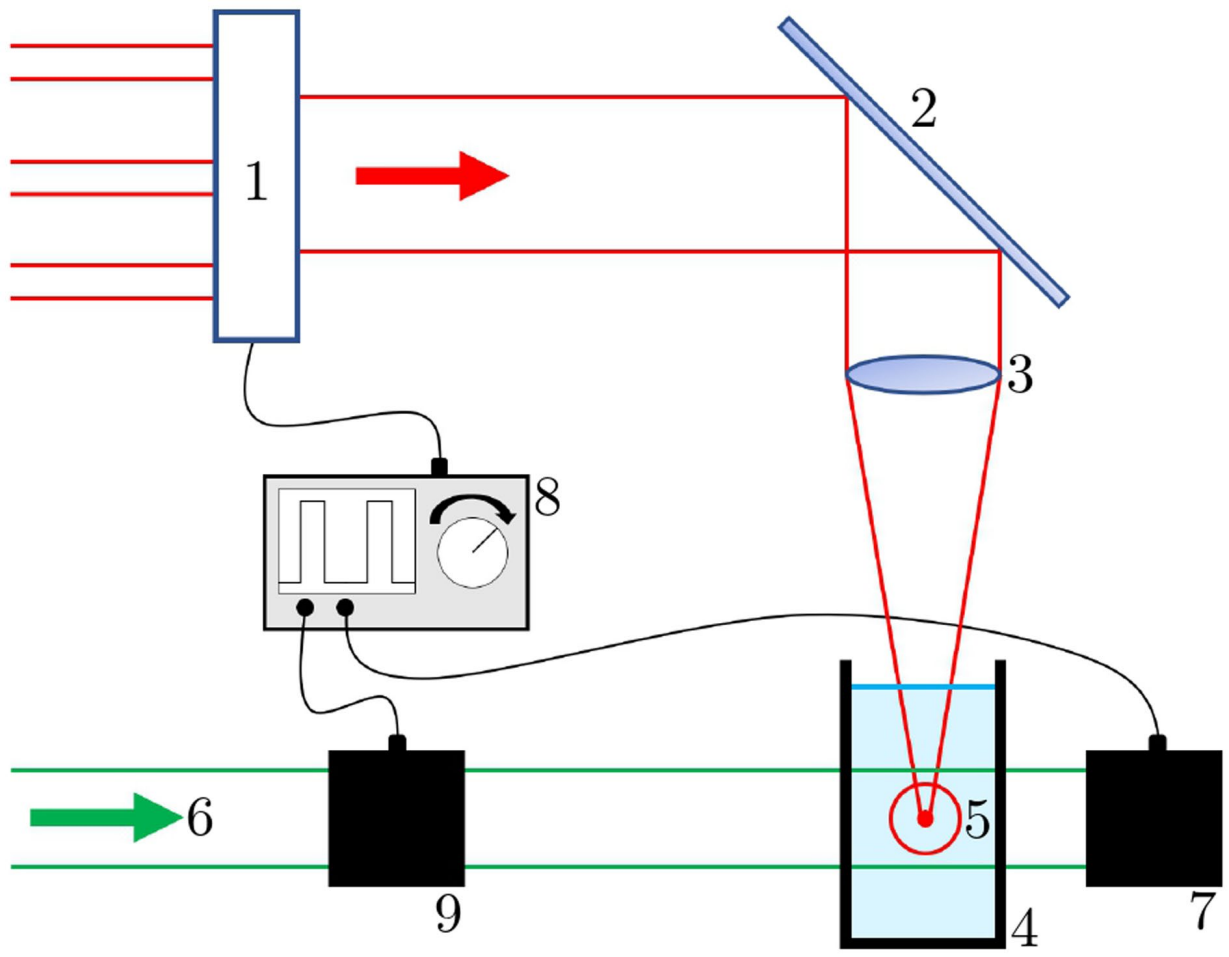
Cavitation bubble measurement set-up. 1: switch for different laser sources; 2: laser mirror; 3: focusing lens; 4: water cuvette; 5: laser breakdown with cavitation bubble; 6: illumination source; 7: camera; 8: function generator; 9: Pockels cell.

For cavitation bubble investigation, the streak camera set-up was modified (see Figure 2): The water bath was replaced by a 2.5 mL polystyrene macro cuvette and the illumination source was replaced by a 1,062 nm cw laser source (Laser 4, see Sec. 2.1), triggered and modulated with a Pockels cell. The camera was changed to the system camera *VEXU-24 M* (*Baumer Electric AG, Frauenfeld, Switzerland*). Both the Pockels cell and the camera are triggered by the waveform generator to the pulse repetition rate of the breakdown laser. This set-up provides a minimum illumination time of 5 ns, which is limited by the switching time of the Pockels cell.

### 2.4 Method for heat investigation

The heat input of the different laser sources was investigated using the thermal imaging camera optris *Xi 400* (*Optris GmbH, Berlin, Germany*). The different lasers were focused via the laser scanner set-up either on black anodized aluminum plates or on porcine eye irises. The scanner scribed a 5 mm long line with 10 mm/s. The emissivity was set to to ε_eye_ = 0.98 for the iris tissue (equal to water and skin) and ε_alu_ = 0.67 for anodized aluminum (38). The measurements are then compared to simulations computed with *MATLAB* (*The MathWorks, Inc., Natick, United States*) following the approach of Ansari et al. (39).

## 3 Results

This section includes results of the different ophthalmic surgeries, shock wave and cavitation bubble examination, and heat investigation.

### 3.1 Laser Iridotomy

**Figure 3:**
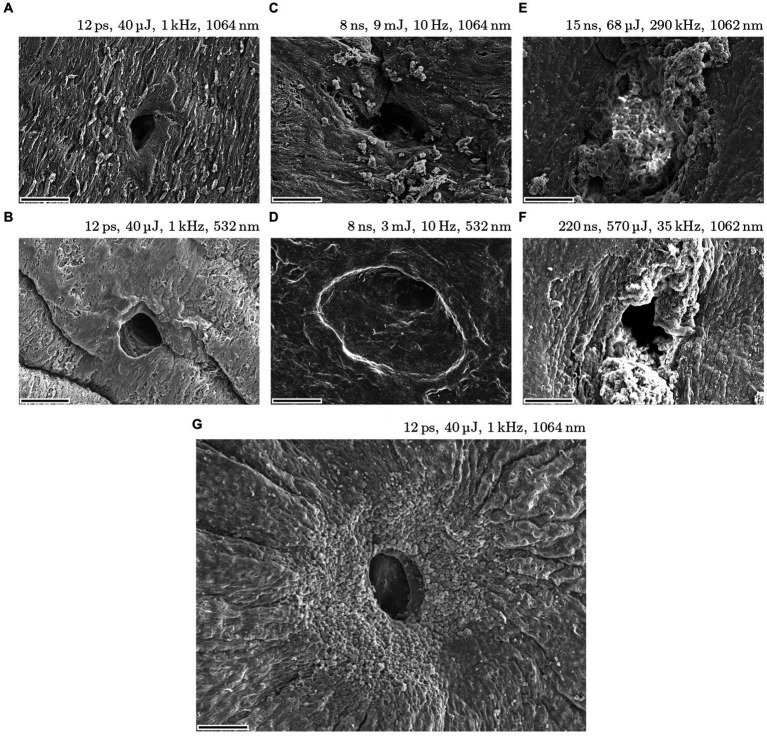
Iridotomies on *post mortem* porcine eye irises with pulse widths of 12 ps, 8 ns, 15 ns and 220 ns. Images **(A–F)** show typical results for each pulse width. Magnification factor is 500 and scale bars are 50 μm. Image **(G)** shows an ideal result for an iridotomy (SEM mag. Factor is 230).

**Figure 4:**
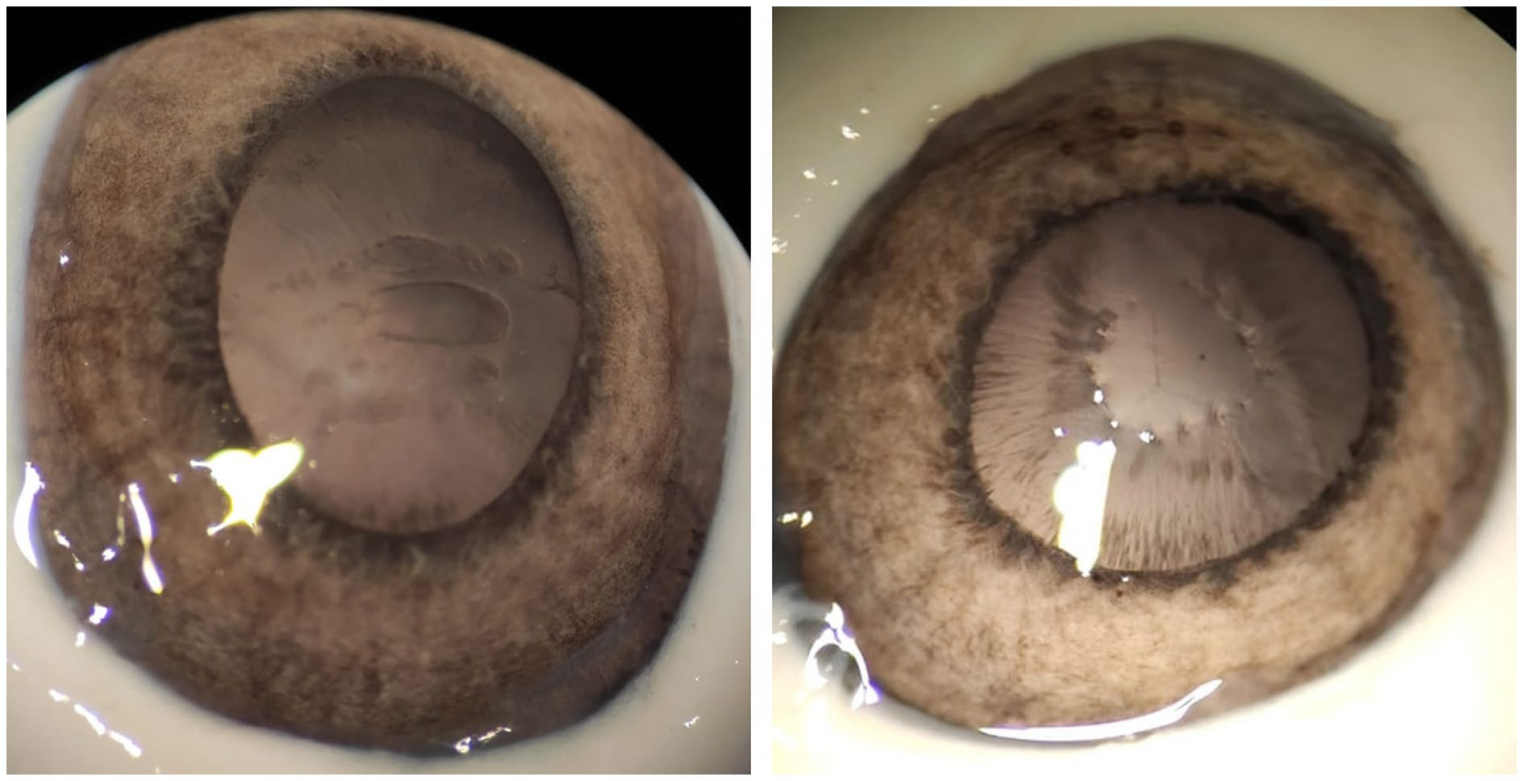
Cuts (left) and piercings (right) in anterior lens capsules of porcine eyes with low-energy 12 ps USP laser pulses.

The study revealed a major variation in size, shape, quality and reproducibility of iridotomies with different pulse widths. A comparison of four different pulse widths (12 ps, 8 ns, 15 ns, 220 ns) is shown in Figure 3. The images show typical results for each pulse width. In each case, the SEM magnification factor is 500 and the scale bars are 50 μm. While results (**A**) and (**B**) for 12 ps, 1 kHz at 1064 nm and 532 nm are very good, showing small and well-defined holes, the nanosecond results (**C**)–(**F**) with different pulse lengths, pulse energies, repetition rates, and wavelengths show frayed edges, ejection of ablated material, and large areas of altered surrounding tissue. The latter is attributed to the higher temperatures incurred through these pulse widths. Furthermore, Figure 4G shows an ideal result, which was achieved with the 12 ps USP laser source, with 40 μJ pulse energy, 1 kHz repetition rate, and 1 s irradiation time. Here, the quality is almost perfect: The ablated hole has very sharp edges and appears almost as if punched. The scale bar is also 50 μm but the SEM magnification factor is 230.

In addition to the far better defined laser processing, the pulse energy necessary for piercing the iris could be reduced to several 10 of microjoules (between 30 μJ and 70 μJ) when using USP laser pulses compared to several millijoules (1 mJ to 10 mJ) when using ns laser sources. To achieve complete penetration of the iris, approximately 1,000 USP pulses, dependent on the pulse energy, are required (and thus an exposure time of 1 s at 1 kHz repetition rate). The ablation per pulse is considerably lower than with nanosecond pulses, where a few pulses (with 5 mJ pulse energy) are sufficient to achieve complete penetration of the iris.

### 3.2 Capsulotomy

With ps pulses, well-defined capsulotomies can be performed in addition to iridotomies. As seen in Figure 4, precise cutting (left) and piercing (right) of the anterior lens capsule of porcine eyes is possible with 12 ps laser pulses—without tearing the remaining capsule. Similar to iridotomy, pulse energies between 30 μJ and 70 μJ prove to be suitable.

**Figure 5:**
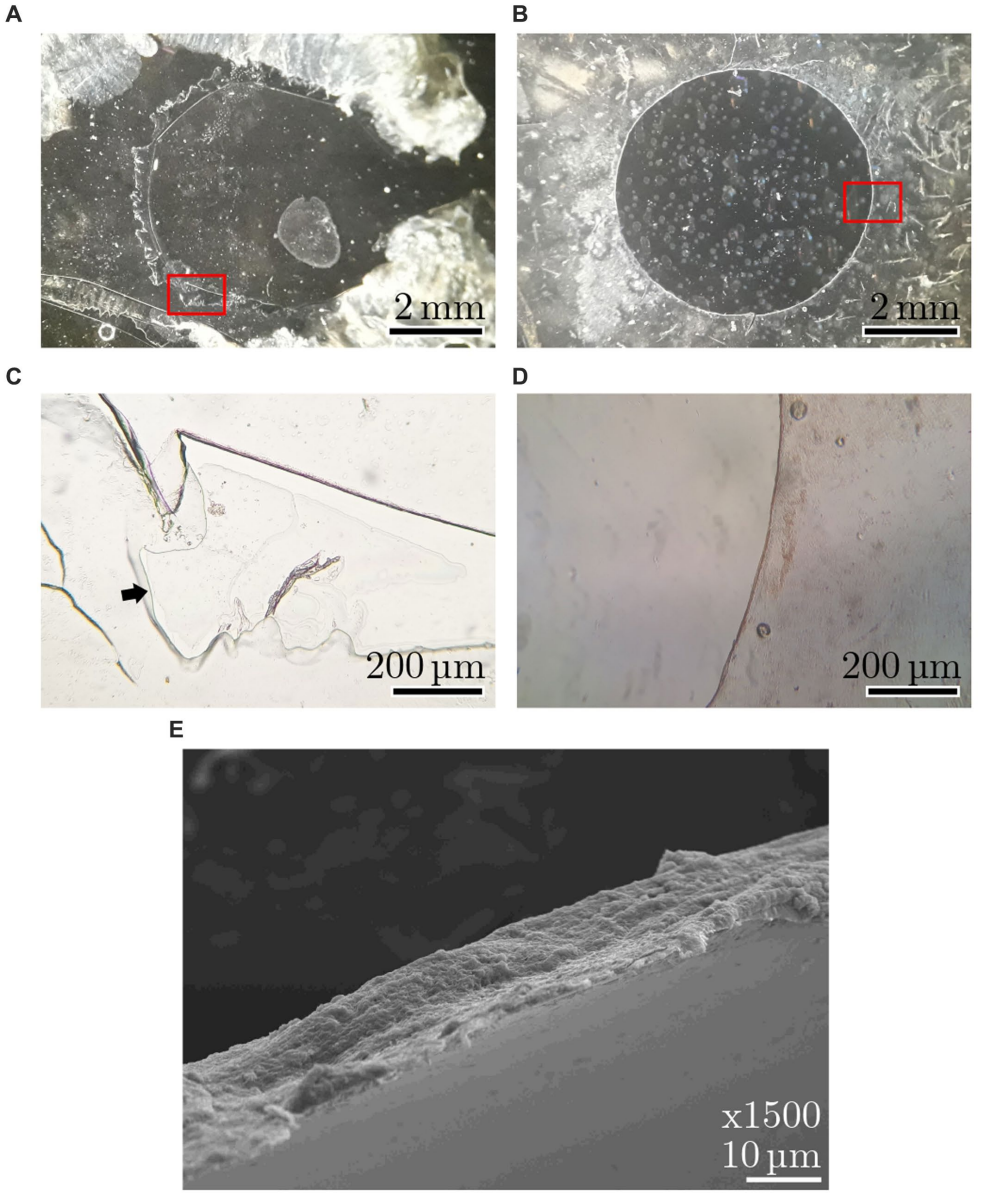
Anterior capsules with 4 mm capsulotomies with **(A)** 8 ns, 3 mJ and **(B)** 12 ps, 50 μJ. Images **(C,D)** show light microscopy magnifications of respective cutting edges [magnified areas are marked in red in **(A,B)**]. The black arrow in **(C)** marks the edge, since it is folded over. Image **(E)** shows an SEM visualization of the cutting edge of the 12 ps, 50 μJ capsulotomy.

Figure 5 shows a comparison of capsulotomies with (**A**) 8 ns, 3 mJ and (**B**) 12 ps, 50 μJ. Both capsulotomies are performed on dissected anterior lens capsules. The lasers were focused on the capsules via the laser scanner head set-up. The capsulotomy diameter was set to 4 mm and the laser processing speed was set to 2.5 mm/s, resulting in a spot spacing of 5 μm for the 1 kHz ultrashort pulse train and 250 μm for the 10 Hz, 8 ns pulse train. Due to high energy input with accompanying high shock impact of the ns laser pulses, the processing speed could not be reduced to achieve the same spot spacing as for the USP pulses, since the capsule would have been torn right away. A comparison of the images reveals a big difference in capsules shape and cut quality. While the ns capsulotomy is torn and has frayed edges due to the high pulse energy required for these pulse widths, the USP capsulotomy is almost perfectly round with sharp and smooth cutting edges. The lower images (**C**) and (**D**) show higher magnifications of the respective capsulotomies cutting edges, visualized by light microscopy. In (**C**) the cutting edge is marked with a black arrow, since the capsule is folded over due to the high shock input. Additionally, Figure 5E shows an SEM visualization of the cutting edge of (**B**) with a magnification factor of 1,500. Here, the edge is very sharp and flat, which is comparable to state-of-the-art femtosecond and manual capsulotomies (19, 23).

### 3.3 SLT—selective laser-trabeculoplasty

**Figure 6:**
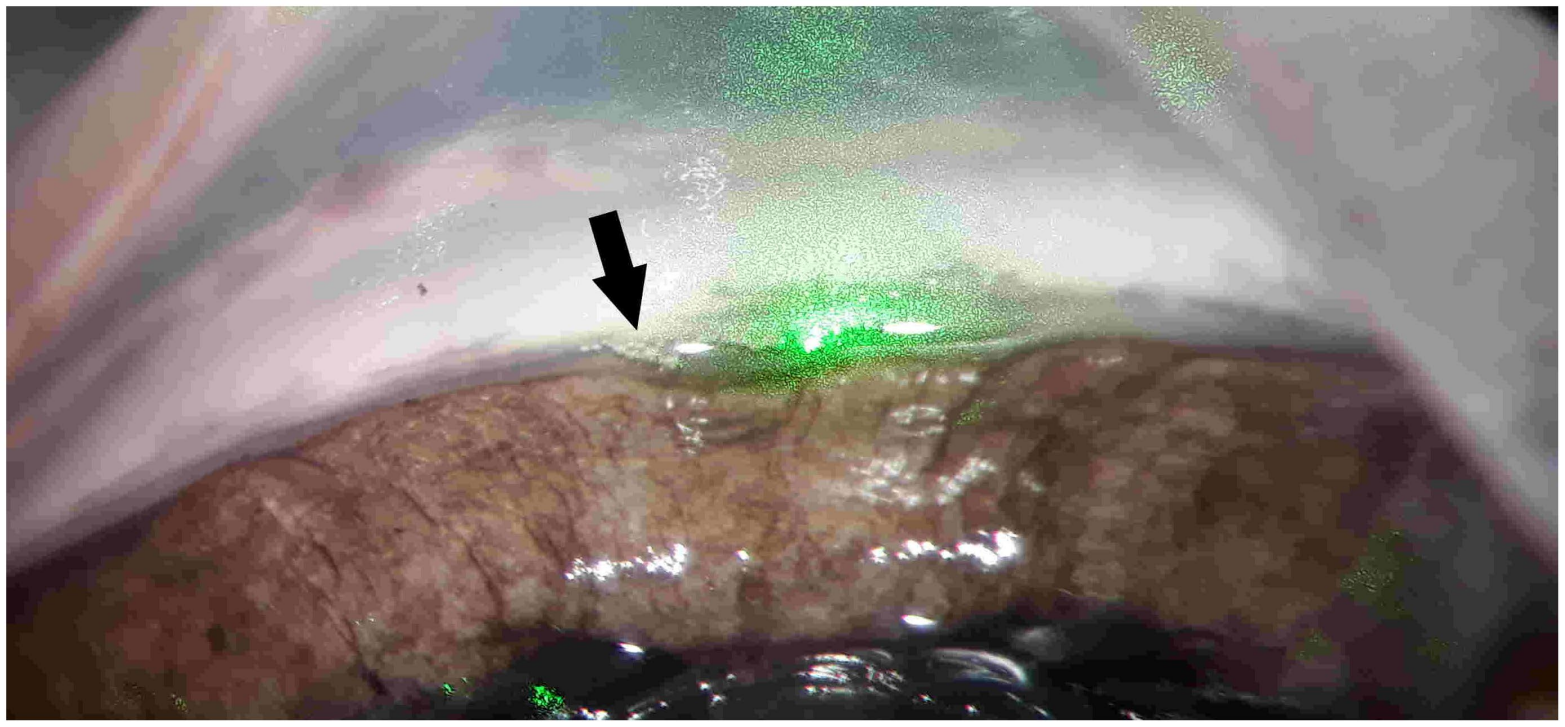
SLT with 1,064 nm, 12 ps and 50 μJ on a bisected porcine eye. “Champagne bubbles” (marked with black arrow) to the left of the green target lasers are considered an indicator of successful treatment.

Similar to the results of the iridotomy and the capsulotomy, SLT is possible with the 12 ps USP laser pulses at much lower pulse energies compared to ns pulses, namely 30 μJ to 70 μJ instead of 1 mJ (40). A result with 12 ps, 50 μJ at 1064 nm is shown in Figure 6, where “champagne bubbles” can be seen to the left of the green target laser (marked with black arrow). These bubbles appeared during the surgery and are considered an indicator of successful treatment. All tested pulse energies between 30 μJ and 70 μJ led to comparable results.

**Figure 7:**
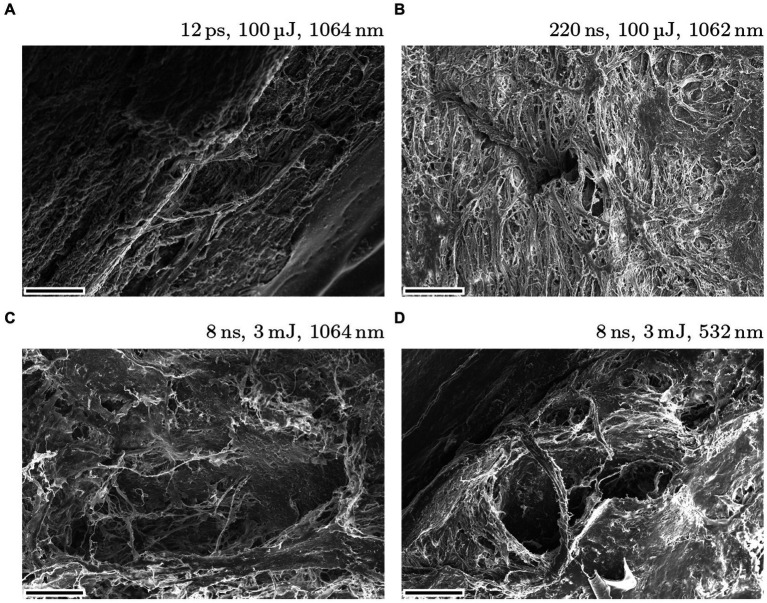
Typical SLT results on exposed porcine eye’s trabecular meshwork using 12 ps USP **(A)** and nanosecond **(B–D)** laser sources. The magnification factor is 200 and the scale bars are 100 μm.

Figure 7 shows four typical results of SLTs in exposed trabecular meshwork with (**A**) picosecond ultrashort laser pulses and (**B**)–(**D**) nanosecond laser pulses. All SEM visualizations were taken with a magnification factor of 200. As with the other surgeries, the differences between USP and ns pulses are significant. Regardless of wavelength and pulse energy, 12 ps produce a small hole with sharp edges and clear boundaries. In contrast, all results with nanosecond pulses show large holes with poor quality. Furthermore, the heat affected zones are strongly pronounced around the holes.

### 3.4 Shock wave and cavitation bubble investigations

**Figure 8:**
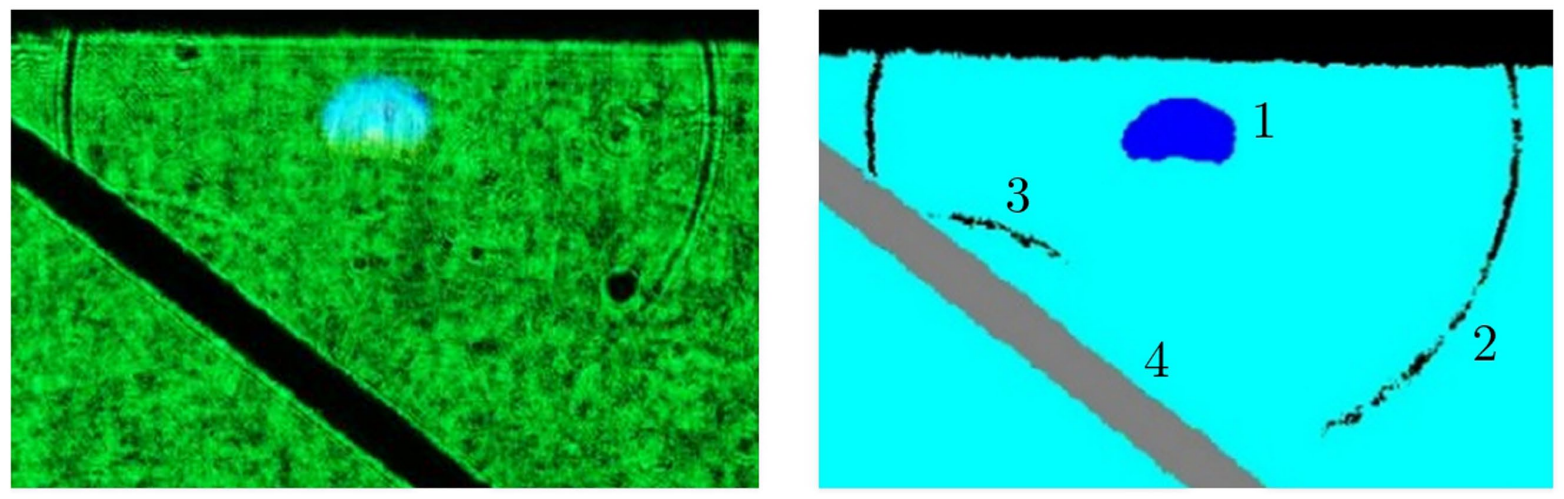
Visualization of a shock wave in water induced with a 147 μJ, 12 ps, 1,064 nm pulse train; left: real image; right: false color representation. 1: Laser induced breakdown; 2: Shock wave; 3: Shock wave reflection; 4: Steel target for reflection. The laser was irradiated from the top (41).

**Figure 9:**
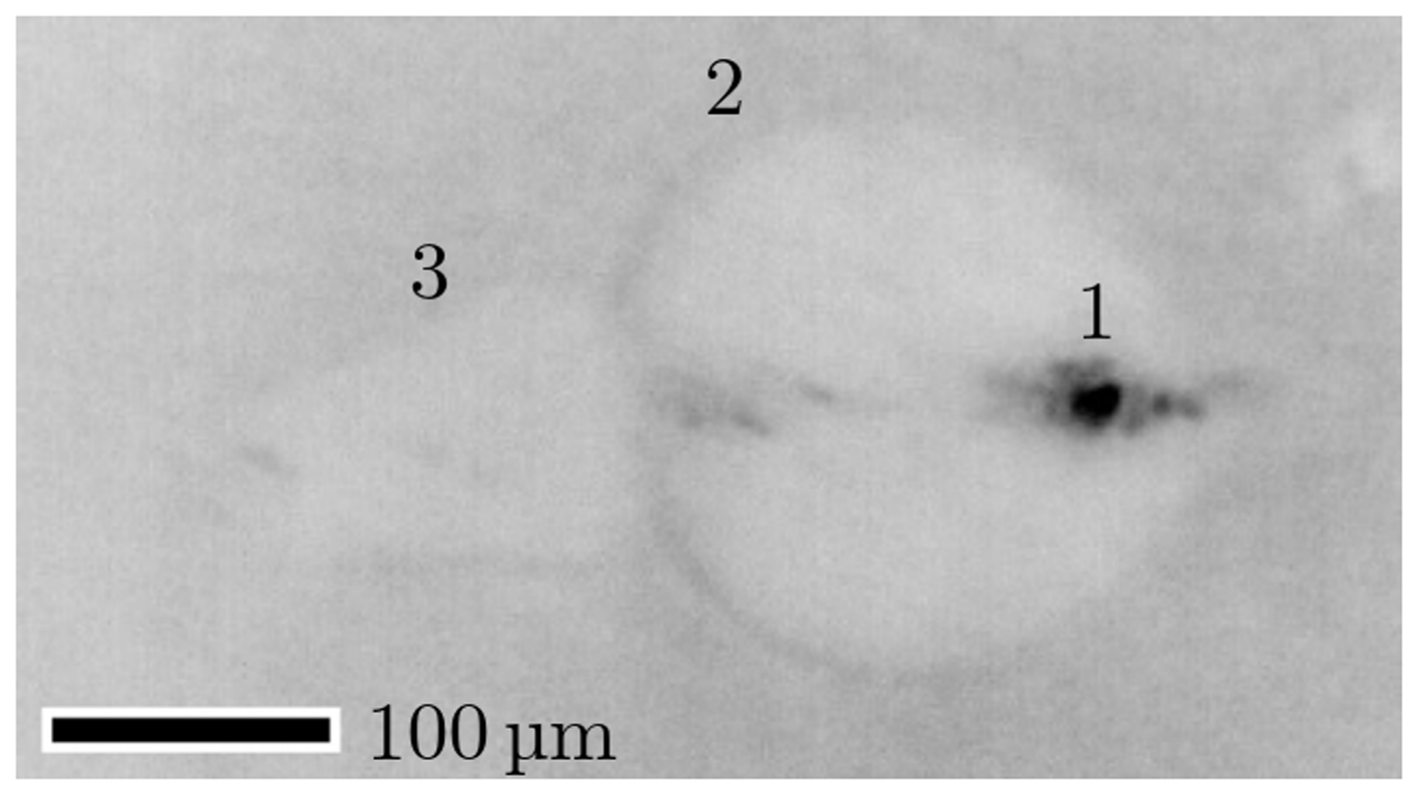
Photograph (inverted colors) of a cavitation bubble with 161 μJ, 12 ps at 1064 nm. The dark spot (1) results from the laser induced plasma and the brighter circle from the cavitation bubble (2). The maximum bubble radius is 198 μm. Moreover, a typical jet formation (3) can be seen left of the bubble. The laser was irradiated from the left (42, 43).

Figure 8 shows the visualization of a laser induced shock wave in water—real image on the left and false color image on the right. The laser induced breakdown leads to a shock wave, which is reflected on a steel target. Here, the breakdown was induced with 147 μJ, 12 ps at 1064 nm from the top. Figure 9 shows a color-inverted photograph of a laser induced breakdown (dark spot) with 161 μJ, 12 ps at 1064 nm together with a cavitation bubble (brighter cycle) and a typical jet formation to the left of the bubble.

**Figure 10:**
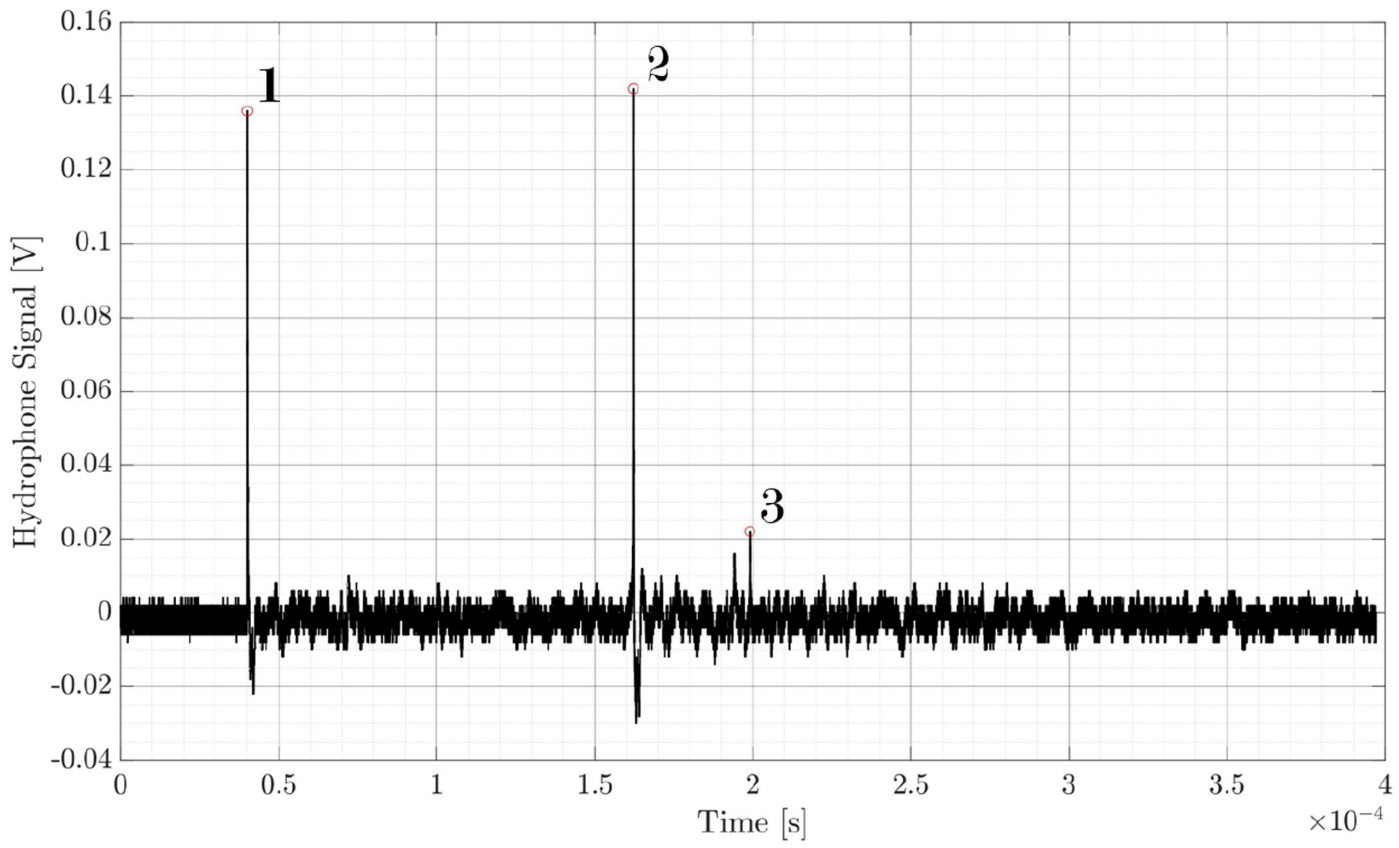
Shock wave pressure measurement with a needle hydrophone. Peak 1: initial breakdown with expansion of the cavitation bubble; Peak 2: first bubble collapse; Peak 3: second bubble collapse. The laser induced breakdown is initiated with 5 mJ pulse energy and 1.6 ns pulse width.

The shock wave pressure was measured with a hydrophone. Figure 10 shows a typical hydrophone measurement visualized with an oscilloscope. Peak 1 is the initial breakdown with expansion of the cavitation bubble—this is the shock wave of the laser induced breakdown (LIB). Peak 2 is the signal from the first bubble collapse and peak 3 from the second bubble collapse. Dependent on the breakdown energy, and therefore on the pulse energy, the cavitation bubble oscillation of expansion and collapse repeats more often. Here, the pulse energy of 5 mJ at a pulse width of 1.6 ns led to three (detectable) shock waves.

**Figure 11:**
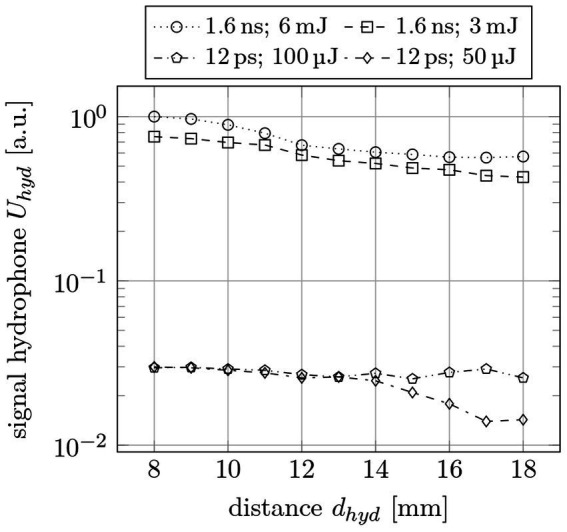
Normalized shock wave signal *U_hyd_* as a function of the hydrophone distance *d_hyd_* for different pulse widths *τ_pulse_* and pulse energies *E_pulse_*.

The needle hydrophone measurements are shown in Figure 11 (logarithmic scale due to major differences in amplitude). Here, the normalized hydrophone signal (voltage) *U_hyd_* is plotted for 12 ps and 1.6 ns pulse width as a function of the pulse energy *E_pulse_* and the hydrophone distance *d_hyd_* to the focal spot. The pulse energies were chosen accordingly to the results from the ophthalmic surgeries, as 50 μJ are typical for USP and 3 mJ and 6 mJ are typical for ns laser sources. 100 μJ at 12 ps are measured to gain information about energy dependency at a fixed pulse width. As can be seen, there are large differences of a factor of 25 in average for ns/USP lasers between the different parameters.

**Figure 12:**
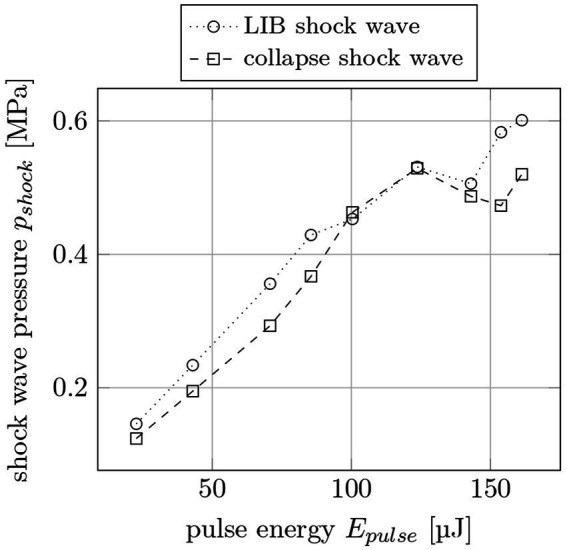
Shock wave pressure *p_shock_* for LIB and collapse shock waves for different pulse energies *E_pulse_* at 6 mm distance to the breakdown. The pulse width is 12 ps.

To determine the actual pressure of the shock waves, the hydrophone voltages are converted to pressure values via spectrum analysis. In Figure 12, the LIB shock wave and the first collapse shock wave pressure are plotted for different pulse energies. The pulse width is 12 ps and the distance between the hydrophone and the breakdown area is 6 mm. This measurement results in higher pressures for higher pulse energies with a maximum pressure of 0.6 MPa at 161 μJ pulse energy.

**Figure 13:**
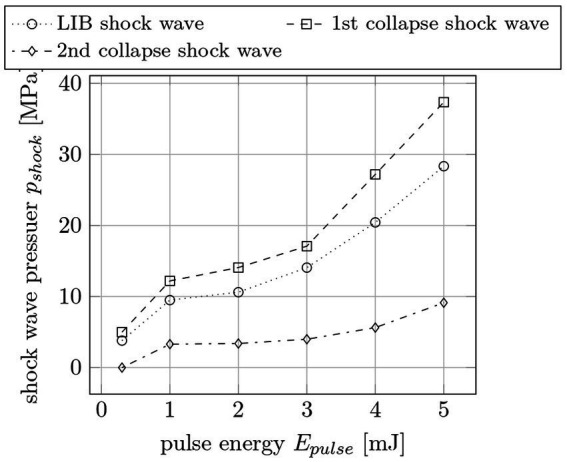
Shock wave pressures *p_shock_* for the first, second and third shock wave per pulse for different pulse energies *E_pulse_* at 17 mm distance to the breakdown. The pulse width is 1.6 ns.

A similar measurement is made for pulse energies between 0.2 mJ and 5 mJ at 1.6 ns pulse width (Figure 13). The distance between the needle hydrophone and the laser induced breakdown is 17 mm. The measurement was done for the LIB, the 1^st^ and the 2^nd^ collapse shock wave. It results in much stronger shock wave pressures compared to 12 ps pulses—even for longer distance. For example, a 50 μJ, 12 ps laser pulse leads to a maximum pressure of 0.25 MPa while a 5 mJ, 1.6 ns laser pulse generates 37 MPa.

### 3.5 Heat investigation

**Figure 14:**
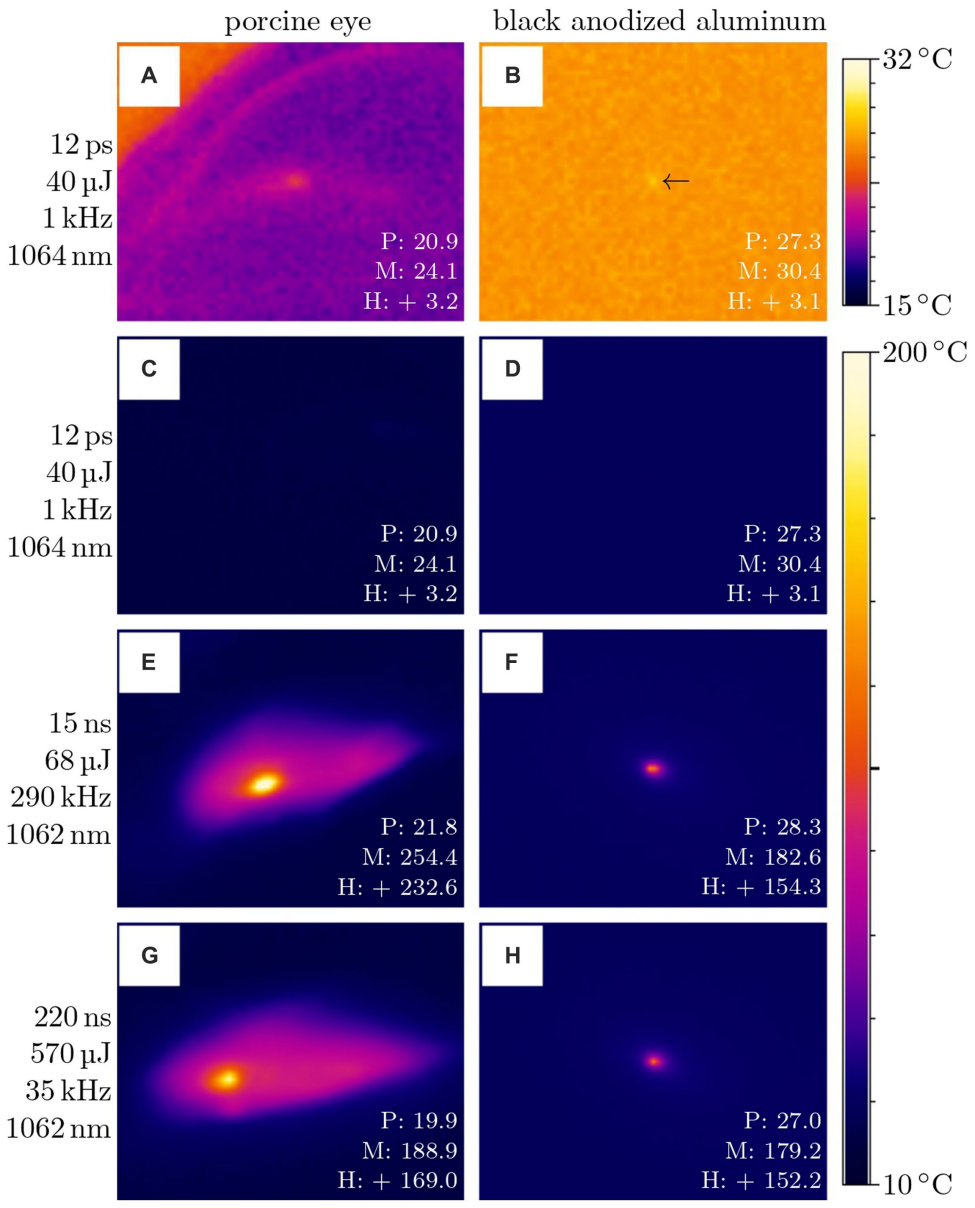
Temperature measurement on porcine eyes **(A,C,E,G)** and black anodized aluminum **(B,D,F,H)** with different laser parameters. The values given are the temperature before irradiation *P*, the maximum temperature during irradiation *M*, and the heating difference *H* in °C. Measurement **(A)** and **(C)** as well as **(B)** and **(D)** are the same, with different temperature scales. The lasers scribed a 5 mm long line with 10 mm/s.

Large differences between treatment with picosecond and nanosecond pulses also exist with respect to heat input and transfer around the laser focus (see Figure 14). On the left, the test is shown on porcine eyes for pulse widths of 12 ps, 8 ns, 15 ns and 220 ns as used for ophthalmic surgeries (Figures 14A,C,E,G), on the right, for comparison on black anodized aluminum (Figures 14B,D,F,H). As mentioned in the methods (see Sec. 2.4), the lasers scribed a 5 mm long line with 10 mm/s. The respective temperature before irradiation *P*, maximum temperature during irradiation *M*, and heating difference *H* in °C are indicated. Results (**A**) and (**C**), as well as (**B**) and (**D**) are the same, with different temperature scales for both better visibility and comparability to the nanosecond results. While 12 ps only lead to a marginal heating of a few degrees which is not even recognizable in the images (**C**) and (**D**), both 15 ns and 220 ns lead to a heating of more than 150°C. Again, the pulse energies for the various pulse widths were selected as required for the treatment.

**Figure 15:**
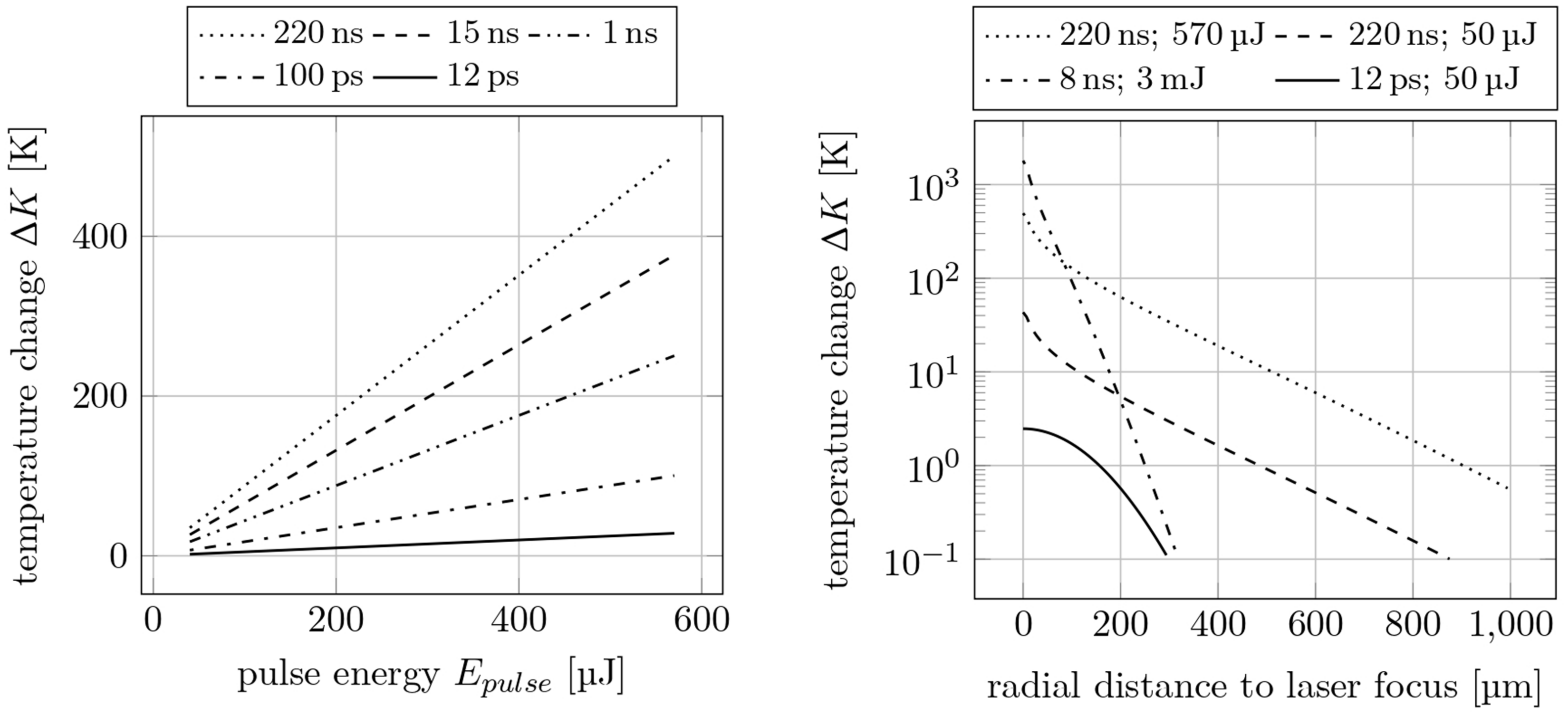
Simulated temperature changes on a water surface. Left: for different pulse widths *τ_pulse_* and pulse energies *E_pulse_*; Right: radial to the laser spot for 220 ns, 8 ns and 12 ps pulse width.

Figure 15 presents simulations of the temperature change on a water surface for different pulse widths. The simulations are calculated for one single pulse each, so that the heat accumulation for several pulses has no effect. As displayed in Figure 15 left (temperature change as function of pulse energy), there is a large difference for pulse widths of 12 ps, 100 ps, 1 ns, 15 ns and 220 ns: The longer the pulse width and the higher the pulse energy, the higher the temperature change. As expected, utilization of low energy ultrashort pulses results in much lower heat inputs than high energy nanosecond pulses (2.5 K change for 12 ps, 50 μJ and 250 K change for 1 ns, 570 μJ). This simulation in general agrees with the experimental results. It should be noted that the simulation reflects the maximum temperature at the center of the laser focus, while the measurement averages over a larger area of space (about 0.15 mm^2^). On the right in Figure 15, the temperature change is plotted as a function of distance to the laser spot. Here, 12 ps, 50 μJ pulses lead to a heat-affected zone of about 300 μm, while it is about 900 μm for 220 ns, 50 μJ and even larger for 220 ns, 570 μJ. Pulses with 8 ns, 3 mJ show a similar heat-affected zone than 12 ps. However, the much higher temperature change of 1800 K (8 ns) compared to 2.5 K (12 ps) would affect the surrounding tissue, e.g., due to the aforementioned stronger shock waves and instant evaporation.

Whereas the impact of a single ps laser pulse is significantly lower compared to nanosecond pulses, the question remains whether or not this holds true for the required pulse train of some hundreds to thousand pulses. The thermo mechanical response of tissue to pulsed irradiation was investigated by, e.g., Vogel et al. (44) and Yakovlev et al. (45). According to these studies typical thermal relaxation times are in the range of a few microseconds. In comparison to the temporal distance of consequent pulses, which is in the millisecond range for ps lasers, this relaxation time ensures temperature and stress confinement in any case.

## 4 Discussion and conclusion

As expected, the use of ultrashort pulses in ophthalmic surgeries led to significant improvements with regard to ablation precision, surgery quality, and impact on surrounding tissue. By using picosecond pulses instead of nanosecond pulses, the pulse energy could be reduced from a few millijoules to 10 of microjoules (an on-average reduction factor of 100). As with femtosecond lasers, this regime is referred to as cold material processing. At a pulse width of 12 ps, all of the aforementioned ocular procedures, i.e., laser-iridotomy, (post-) cataract treatment/capsulotomy, and selective laser-trabeculoplasty, could be successfully performed in an energy range of 30 μJ to 70 μJ at both 532 nm and 1,064 nm. Furthermore, the shock wave and cavitation bubble formation during the surgeries could be reduced by a factor of x25 on average due to the shorter pulse widths and lower pulse energies. Finally, the thermal studies (experimental and simulative) revealed a significant reduction in both the temperature change and the temperature spreading (spatially and temporally) between picosecond and nanosecond laser pulses.

The recent availability of ultra-compact picosecond lasers—due to new physical approaches in laser physics—could lead to smaller and simpler laser sources. Therefore, the size- and cost-related disadvantages of former picosecond lasers are counteracted, as these systems consist of a two-stage set-up with two different laser resonators. The new approach is just one single micro-chip resonator with (if needed) subsequent pulse compression and/or pulse amplification (24, 25). Thus, the new technology is much simpler than existing systems, leading to a cost reduction and prices comparable to that of state-of-the-art ophthalmic nanosecond lasers.

Compared to former results with picosecond laser sources, we achieved at least comparable, mostly better results. Now it must be confirmed whether these results are also possible with the novel picosecond laser sources, which will be the next step in our project-plan. Furthermore, all results must then be transferred to human tissue *ex vivo*, in order to test the ablation possibilities as closely as possible to *in vivo* samples.

In conclusion, the use of picosecond ultrashort pulse laser sources in ophthalmology leads to substantial improvements and could be the stable basis for a very precise, universal tool for current ophthalmic surgeries and new surgical methods. Therefore, the next step will be to investigate the transferability of results from animal to pathological human tissue, and eventually a clinical study.

## Data availability statement

The original contributions presented in the study are included in the article/supplementary material, further inquiries can be directed to the corresponding author.

## Ethics statement

Ethical approval was not required for the study involving animals in accordance with the local legislation and institutional requirements because the used porcine eyes were exclusively from slaughterhouse waste.

## Author contributions

MiK: Conceptualization, Formal analysis, Investigation, Methodology, Visualization, Writing – original draft. JF: Resources, Writing – review & editing. MF: Investigation, Resources, Writing – review & editing. AG: Investigation, Resources, Writing – review & editing. KK: Funding acquisition, Resources, Writing – review & editing. DK: Funding acquisition, Resources, Writing – review & editing. MaK: Conceptualization, Funding acquisition, Methodology, Supervision, Validation, Writing – review & editing. FL: Formal analysis, Investigation, Methodology, Resources, Validation, Writing – review & editing. JS: Funding acquisition, Methodology, Supervision, Validation, Writing – review & editing. JW: Investigation, Writing – review & editing. BB: Conceptualization, Funding acquisition, Methodology, Project administration, Supervision, Validation, Writing – review & editing.
